# Levosimendan preconditioning in patients undergoing elective cardiac surgery with poor ejection fraction. preliminary results.

**DOI:** 10.1186/1749-8090-10-S1-A310

**Published:** 2015-12-16

**Authors:** R Ávalos, R MartinezSanz, JJ Jiménez, JL Iribarren, J Montoto, A Lacruz, M Brouard, P Garrido, PC Prada, Jorge P Pérez, M García-González

**Affiliations:** 1 Complejo Hospitalario Universitario de Canarias, Tenerife, Spain; 2 Universidad de La Laguna, Tenerife, Spain

## Background and Aims

Left ventricular systolic dysfunction (LVSD) represents a risk factor for the development of low cardiac output syndrome (LCOS) resulting in a high mortality in patients undergoing cardiac surgery, Levosimendan is a drug with inotropic, vasodilatory and organ-protective properties, often used in heart failure and low cardiac output patients.

We study the effects of preoperative levosimendan administration (PLA) in patients with LVSD undergoing elective cardiac surgery on LCOS development and other secondary post-surgery outcomes

## Methods

We retrospectively studied a cohort of patients with LVEF ≥ 45% undergoing elective cardiac surgery from January 2006 to December 2013. Patients who received PLA (infusion of 0.05 to 0.2 mcg /kg /min for 24 hours without loading dose) - (Group I) within 72 hours prior to surgery were compared with those who did not receive it (Group II). Demographic, clinical, hemodynamic, operative characteristics and postoperative outcome were analyzed. PLCO was consider as cardiac index lower than 2.2 l/min/m^2^, without low blood volumen.

## Results

146 patients with LVSD were included. 80% were male; mean age 66 ± 9.7 years; LVEF 36 ± 5%; and Euroscore 8.7 ± 8.6 were studied. Group I included 13 and Group II 123 patients.

Among both groups there were no significant differences in age, sex, cardiovascular risk factors, preoperative functional class, LVEF and operative characteristics.

Group I patients had a lower incidence of LCOS (7.7 vs 43.6%; p = 0.012); higher cardiac index (3.2 ± 0.7 vs 2.7 ± 0.8 L/min/m2; p = 0.02); lower troponin I peak levels of (1.9 ± 1.8 ng/mL vs 10.4 ± 32; p = 0.02); lower creatinine peak levels (0.98 ± 0.4 vs 1.3 ± 0.7 mg/dL; p = 0.03 and shorter mechanical ventilation (4 [2-7] vs 6 [5-19] h; p 0.007)). They needed lower maximum dose of dobutamine (1.4 ± 1.9 vs 4.7 ± 5.3 mcg/kg/min; p = 0.016). De novo atrial fibrillation incidence was similar in both groups (30 vs 32%; p = NS). Group I patients had a lower but not significantly postoperative hospital stay (7 [6-10] vs 9 [7-17] days; P = 0.08). There was no differences in mortality at 30 days (0 vs 7.5%; P = NS). After adjusting for preoperative LVEF and NYHA, patients in Group I showed less risk of LCOS development (OR: 0.11; 95% CI [0.01-0.85]; p = 0.005).

## Conclusions

preoperative administration of Levosimendan reduces the incidence of LCOS and exerts beneficial effects on myocardial and renal preservation in high-risk patients with LVSD undergoing elective cardiac surgery.

